# Effects of n-3 Polyunsaturated Fatty Acid Supplementation in the Prevention and Treatment of Depressive Disorders—A Systematic Review and Meta-Analysis

**DOI:** 10.3390/nu13041070

**Published:** 2021-03-25

**Authors:** Maike Wolters, Annkathrin von der Haar, Ann-Kristin Baalmann, Maike Wellbrock, Thomas L. Heise, Stefan Rach

**Affiliations:** Leibniz Institute for Prevention Research and Epidemiology—BIPS, Achterstraße 30, 28359 Bremen, Germany; anvonder@uni-bremen.de (A.v.d.H.); annkristin1497@gmail.com (A.-K.B.); mwellbro@uni-mainz.de (M.W.); heise@leibniz-bips.de (T.L.H.); rach@leibniz-bips.de (S.R.)

**Keywords:** n-3 polyunsaturated fatty acids, eicosapentaenoic acid, docosahexaenoic acid, depression, depressive symptoms, meta-analysis

## Abstract

N-3 polyunsaturated fatty acids (PUFAs) have been suggested to affect depressive disorders. This review aims to determine the effect of n-3 PUFAs on depressive symptoms in people with or without diagnosed depression. Medline, PsycINFO, and Cochrane CENTRAL databases were searched for randomized controlled trials (RCTs) assessing the association between n-3 PUFAs and depressive symptoms or disorders as outcomes. A random-effects meta-analysis of standardized mean difference (SMD) with 95% confidence intervals (CI) was performed. Twenty-five studies (7682 participants) were included. Our meta-analysis (20 studies) indicated that n-3 PUFA supplementation lowered depressive symptomology as compared with placebo: SMD = −0.34, 95% CI: −0.55, −0.12, *I*^2^ = 86%, *n* = 5836, but a possible publication bias cannot be ruled out. Subgroup analyses indicated no statistically significant difference by treatment duration of <12 vs. ≥12 weeks, presence of comorbidity, or severity of depressive symptoms. Nevertheless, beneficial effects were seen in the subgroups of studies with longer treatment duration and with no depression and mild to moderate depression. Subgroup analysis by eicosapentaenoic acid (EPA) dosage revealed differences in favor of the lower EPA dosage. Sensitivity analysis including studies with low risk of bias seems to confirm the overall result. Supplementation of n-3 PUFA appears to have a modest beneficial effect on depressive symptomology, although the quality of evidence is still insufficient.

## 1. Introduction

Depressive symptoms are associated with reduced quality of life, increased morbidity and mortality, and rising utilization of the healthcare system [1]. With more than 264 million people affected, depressive disorders are posing a global challenge to finding appropriate prevention measures and treatments for the disease [2].

Although evidence shows that pharmacological interventions are mostly effective for the improvement of depressive symptoms, there is existing evidence of severe side-effects such as suicidal tendencies, liver damage, or poor compatibility with other drugs, each of which can cause lower adherence to antidepressants [3,4,5]. Hence, there is a necessity for the development of better-tolerated therapies for patients with depressive symptoms or disorders, as individual or adjuvant therapy.

N-3 polyunsaturated fatty acids (n-3 PUFAs) are components of cell membranes and are essential for many aspects of physiological function, such as the general brain metabolism and, in particular, neuronal processes associated with depressive symptoms [6,7,8]. Several potential mechanisms have been suggested for the antidepressive effects of n-3 PUFAs. They seem to beneficially influence monoamine neurotransmission, neurogenesis, and inflammatory responses [9]. The role of n-3 PUFAs in inflammation has been confirmed by an RCT in adults with high or low inflammatory status, which indicated that supplementation of eicosapentaenoic acid (EPA) resulted in a relevant decrease in depression scores only in those with high inflammatory status [10]. Preclinical results also suggest a role of n-3 PUFAs in the function of the hypothalamic–pituitary–adrenal (HPA) axis. Docosahexaenoic acid (DHA) supplementation has been shown to decrease the levels of HPA hormones, and DHA levels were inversely associated with hypercortisolemia [9]. Nevertheless, results of previous studies suggested that EPA may be more effective in the treatment of depression [9].

There are plausible biological explanations why a low n-3 PUFA status may lead to mood disorders, such as depression [11]. Accordingly, numerous studies on n-3 PUFA levels in blood found links between depressive symptoms and low EPA and DHA status [12,13,14,15,16].

Hence, much research has focused on investigating the efficacy of n-3 PUFA in treating depression in randomized controlled trials (RCTs). Previous systematic reviews and meta-analyses showed mixed effects of n-3 PUFA supplementation on depressive symptoms [17,18,19,20,21,22]. Some did, however, express scepticism regarding the quality of evidence of included RCTs and possible publication bias [23]. Since then, new relevant RCTs have been published (e.g., [24,25]), allowing for a reassessment of the findings concerning the efficacy of n-3 PUFA and the quality of included RCTs. Two recent systematic reviews published in 2019 differed from our approach: one included adults with a diagnosis of clinical depression only [18], and the other included studies in adults with a minimum treatment duration of 24 weeks only [17]. The first reported an overall beneficial effect of n-3 PUFAs on depression, whereas the latter found that supplemental n-3 PUFA intake probably had little or no effect on the risk or development of depressive symptoms. We were interested in the effects in prevention and treatment of depression, as well as in shorter treatment duration, as some trials also found n-3 PUFA effects after 12 [26] or even after 8 weeks [27].

Therefore, this review and meta-analysis also includes studies with nondepressed participants and with shorter treatment durations. It aims to determine the effect of n-3 PUFA supplementation in the prevention or treatment of depressive symptoms including recent relevant studies in order to provide evidence-based recommendations.

## 2. Materials and Methods

### 2.1. Search Strategy and Study Eligibility

The current systematic review was conducted in accordance with the Preferred Reporting Items for Systematic reviews and Meta-Analysis (PRISMA) guidelines [28]. A.K.B. and an experienced librarian searched Medline via Ovid, PsycINFO via Ovid, and CENTRAL via the Cochrane Library for studies published between January 2010 and 1 January 2020 using a combination of subject and free-text terms with no language restriction. The searches combined terms related to (i) n-3 PUFA supplementation such as “n-3 PUFA”, “eicosapentaenoic acid”, “docosahexaenoic acid”, and “fish oil”, and (ii) depressive symptoms such as “depression” and “major depressive disorder”; the results were filtered to include studies in humans only. Studies were excluded if they investigated special types of depression (e.g., perinatal depression, bipolar depression, and alpha-interferon-induced depression) or depression in combination with psychotic or neurodegenerative diseases. No restrictions were applied regarding age of the study participants or treatment duration.

Furthermore, reviews, meta-analyses, observational studies, articles with insufficient information, guidelines, dissertations, editorials, case reports, and unpublished studies were not considered. Details on the search strategy (Medline, PsycINFO, and Cochrane CENTRAL) are provided in Table S1 (Supplementary Materials).

On the basis of the inclusion and exclusion criteria, two authors (A.K.B., M.We.) independently screened titles, abstracts, and full texts of articles for eligibility. Disagreement in two cases was resolved by reaching consensus and by consulting a senior author (M.Wo.). Moreover, recent relevant systematic reviews and meta-analyses that were identified through forward searching (March 2020) were searched for eligible RCTs [17,18,23,29,30]. If there were multiple reports of the same study, the report first published providing all relevant outcome data and study characteristics was considered in this review.

### 2.2. Data Extraction

Using a predefined data extraction form, two authors (A.K.B. and A.v.d.H.) independently extracted all relevant study characteristics including country, assessment methods, number and characteristics of participants, type of n-3 PUFA, follow-up time, and outcome data. A third author resolved disagreement if needed (M.Wo.). Corresponding study authors were contacted for missing relevant information. The overview on the included studies followed the PICO criteria (population, intervention (or exposure), comparison (if applicable), outcome). If depressive symptomology was assessed using several scales, data for all scales were extracted.

### 2.3. Assessment of Risk of Bias

Quality assessment of the included RCTs was conducted using the Cochrane Collaboration’s risk of bias tool [31] which includes the evaluation of selection, performance, detection, attrition, and reporting bias of each study and allows judgement as high, low, or unclear risk. The overall quality based on the risk of bias assessment was converted to AHRQ (Agency for Healthcare Research and Quality) standards and was judged as good, fair, or poor [32]. A study was considered to have low risk of bias and good quality if all criteria were met, thus having a low risk of bias for each domain. Fair quality was assumed if one criterion was not met or two criteria were unclear, but only if this was unlikely to have biased the outcome, and if there was no important limitation that could invalidate the results. A study was considered to have high risk of bias and poor quality if two or more criteria were not met or were unclear and if this was likely to have biased the outcome or if there were important limitations that could invalidate the results.

Two authors independently assessed the risk of bias of individual studies (A.v.d.H. and M.We. or M.Wo.), and any differences in quality assessment results were resolved through consensus.

### 2.4. Data Synthesis and Analysis

The analysis was conducted with the most commonly used scale in all studies, according to the following hierarchy: Beck Depression Inventory (BDI) [33], Hamilton Depression Rating Scale (HDRS) [34], Montgomery Asberg Depression Rating Scale (MADRS) [35], Hospital Anxiety and Depression scale (HADS) [36], and others as used in the studies. Continuous data (*N*, mean and standard deviation) were collected per intervention group at baseline and at the end of each intervention. Additionally, change from baseline per group was collected if available. If pooling was meaningful, we undertook meta-analyses of continuous data as standardized mean difference (SMD) with 95% confidence interval (CI) including only the relevant arms in the case of studies with multiple trial arms. Intention-to-treat (ITT) data were used if available; otherwise, data from per protocol populations were included. Study authors were contacted and requested to provide required data if they were not provided in the publication or to verify whether unadjusted ITT-based data were reported in the study. Because of the expected heterogeneity between studies, a random-effects model was applied. *I*^2^ statistics were used to assess heterogeneity, categorized as low (*I*^2^ ≤ 25%), moderate (25% < *I*^2^ <75%), or high (*I*^2^ ≥ 75%) [37].

Duration of intervention (<12 vs. ≥ 12 months), presence of comorbidity (no, yes, both), severity of depression (no/mild to moderate, major, any severity of depression), and dosage of EPA (≥1000 mg/day vs. <1000 mg/day) were prespecified as characteristics for assessment of heterogeneity and were evaluated using subgroups to investigate possible effects due to these differences. In studies with multiple treatment groups with different EPA dosages, the treatment groups were included independently only for subgroup analysis by EPA dosage. For analysis, the same comparator was used for all treatment groups and the *N* from comparison groups was split as equally as possible across the treatment groups.

Sensitivity analyses were conducted including only studies with low risk of bias (high quality), i.e., only those judged as high and fair quality and only those with ITT estimates. Additionally, a leave-one-out sensitivity analysis was performed by removing one study at a time to confirm that the results were not influenced by any single study. Potential publication bias was explored using funnel plot asymmetry [38].

Characteristics and results of the trials that could not be quantitatively pooled were narratively summarized.

## 3. Results

### 3.1. Search Results and Characteristics of the Studies

**Figure 1:**
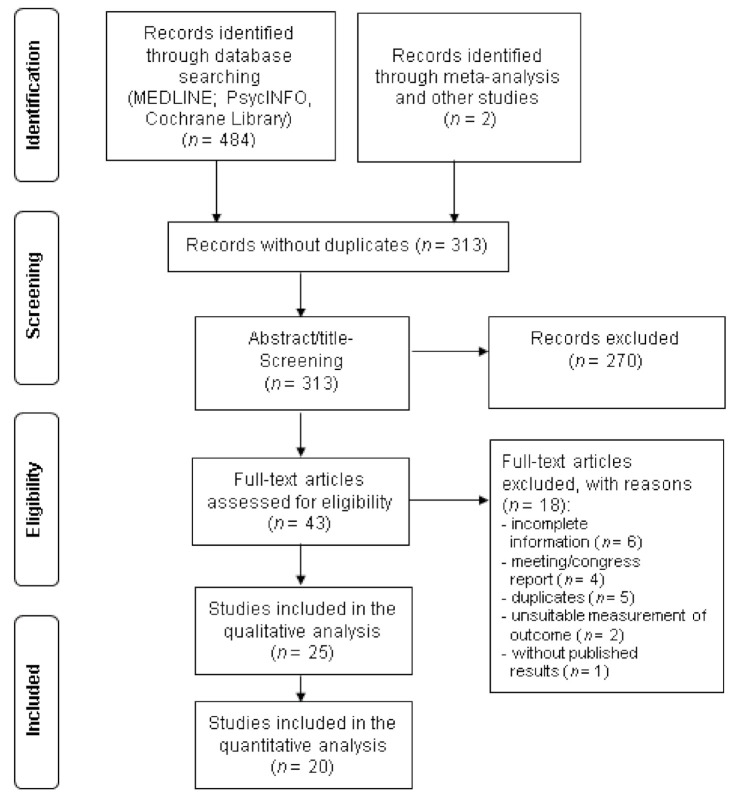
Flow chart of the selection process of randomized controlled trials included in the review.

Twenty-five studies with a total of 7682 participants were included in this systematic review. Figure 1 shows the flow diagram of the screening and selection process. Characteristics of the included studies are shown in Table 1. One of the studies evaluated the effect of n-3 PUFA in healthy women without depression [39] and four studies [40,41,42,43] included participants both with and without depression, while all other RCTs (*n* = 20) considered the effect in the treatment of patients with depressive symptoms.

In 24 of the studies, capsules with EPA and/or DHA were given to participants, five of the studies were explicitly based on fish oil [43,47,48,49,50], and one study used capsules containing alpha-linolenic acid (ALA) only or ALA combined with EPA and DHA in two of the three treatment arms [51]. All studies except one [41] compared the intervention with a placebo treatment. Mostly, n-3 PUFA was examined as a monotherapy for improving depressive symptoms, and two studies used n-3 PUFA as a combination therapy with antidepressant drugs [24,26], whereas, in the other studies, antidepressant standard therapy was maintained if taken by participants. Three studies combined n-3 PUFA with a psychoeducation leaflet [39,52] and/or a stress management program [39] or with a multidomain intervention [40]. The daily dosage of n-3 PUFA ranged from 600 mg to 3600 mg within the studies.

Reported relevant outcomes and the results of the included trials are shown in Table S2 (Supplementary Materials). In 14 RCTs, the BDI was applied as the instrument to evaluate the outcome, six studies applied the HDRS, five studies used the Geriatric Depression Scale (GDS) [53], four studies used the MADRS, and one study used the Children’s Depression Inventory (CDI) [54]. Ten RCTs used more than one assessment to determine depressive symptoms.

Treatment duration varied from 3 to 160 weeks. Total sample sizes ranged from 21 to 4116 participants. Geographically, eight studies were conducted in North America, seven studies in Europe, six studies in the Middle East, three in Asia, and one in Australia.

Most RCTs included male and female participants. Two studies assessed only female [39,48] and one study assessed only male participants [55]. Two studies were conducted in children and/or adolescents [25,56]. The mean age of participants at baseline in the included studies varied from 15.60 to 83.95 years. In most studies, participants suffered from one or more comorbidities such as cardiovascular diseases (seven studies), diabetes mellitus type 1 or 2 (two studies), multiple sclerosis (one study), hypertension (one study), stroke (one study), thyroid dysfunctions (one study), being human immunodeficiency virus (HIV) positive (one study), and end-stage renal diseases (one study). Moreover, the depression severity ranged from mild to major within the included studies. Ten RCTs were conducted to determine the effects of n-3 PUFA in participants with a major depressive disorder (MDD), nine studies included nondepressed or mild to moderate depressed participants, and five included participants with differences in symptom severity or disease progression. Table 1 summarizes the characteristics of the study populations of the included studies.

The data of 20 studies were included in the meta-analysis, while five studies [40,41,42,43,57] were only included in narrative synthesis (Figure 1). Five of the studies included in the meta-analysis reported per protocol data only [27,43,50,51,55].

### 3.2. Risk of Bias

Almost half of the 25 RCTs included (44%, *n* = 11) were judged as studies with good quality, i.e., as having a low summary risk of bias. Fourteen studies were judged as fair (*n* = 9) or poor (*n* = 5) quality, i.e., as having a moderate or high summary risk of bias, respectively (Table 2). In many studies judged as poor or fair quality, the domain “incomplete outcome” was judged with unclear or high risk of bias because of a high dropout proportion and/or per protocol analysis only.

### 3.3. Effects of n-3 PUFA in Meta-Analysis

**Figure 2:**
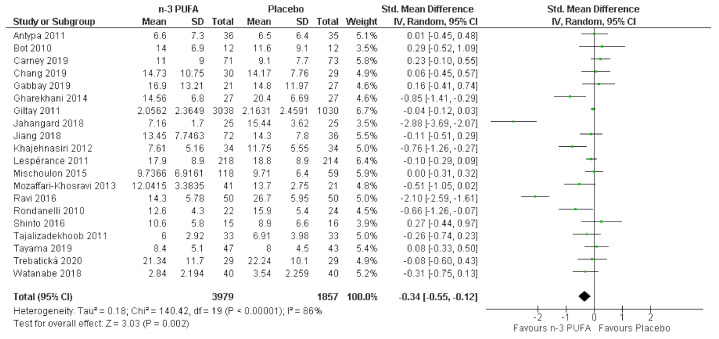
Forest plot of the comparison of the effect of n-3 PUFA vs. placebo on depressive symptoms.

**Figure 3:**
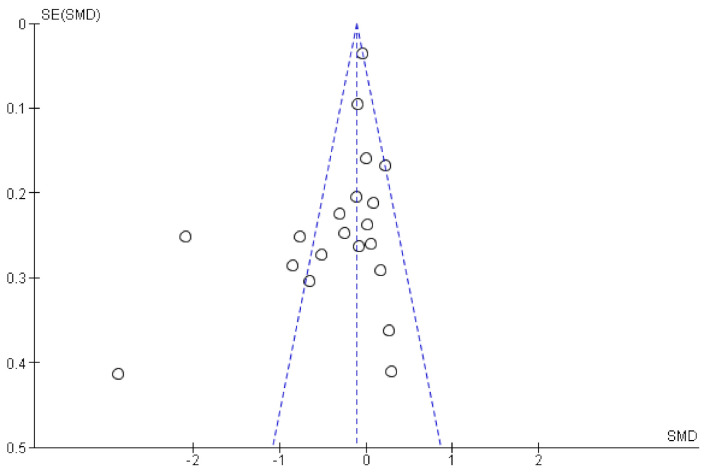
Funnel plot of the comparison of the effect of n-3 PUFAs vs. placebo on depressive symptoms.

Twenty studies including 5836 individuals were included in the analyses. Results are reported as standardized mean difference (95% CI) and showed that n-3 PUFA supplementation lowered depressive symptomology as compared to placebo: SMD = −0.34, 95% CI: −0.55, −0.12. However, there was substantial heterogeneity between studies (*I*^2^ = 86%). The effect sizes were small or modest (Figure 2). Asymmetries in the funnel plot (Figure 3) suggest that results might be influenced by biasing factors such as publication bias. Two studies are particularly responsible for this asymmetry [26,27] as they showed the strongest beneficial effects of supplemental n-3 PUFA intake.

### 3.4. Subgroup Analyses

**Figure 4:**
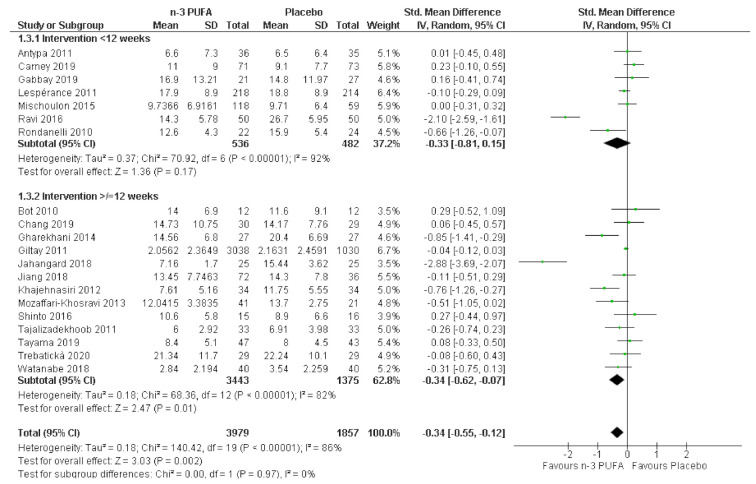
Effect of n-3 PUFAs on depressive symptoms by subgroups of studies with treatment duration of <12 vs. ≥12 weeks.

A recent meta-analysis assumed that beneficial effects may only occur after long-term supplementation and excluded RCTs of less than 6 months duration [17]. In our study, beneficial effects of n-3 PUFAs were observed in the subgroup of studies with longer treatment duration of ≥12 weeks (SMD = −0.34, 95% CI: −0.62, −0.07, *I*^2^ = 82%), as well as in studies with treatment durations <12 weeks (SMD = −0.33, 95% CI: −0.81, 0.15, *I*^2^ = 92%), although, in the latter group, these effects were not statistically significant. There was considerable heterogeneity in both subgroups that requires further exploration. There was, however, no evidence for a statistically significant difference between treatment duration subgroups (chi^2^ = 0.00, df = 1, *p* = 0.97, *I*^2^ = 0%; see Figure 4).

In our analyses, no differences were evident between subgroups of studies including participants “with”, “with/without”, and “without” possible comorbidities (chi^2^ = 2.42, df = 2, *p* = 0.30) and heterogeneity was low (*I*^2^ = 17.3%). No statistically significant effect was seen in any of the three subgroups, with moderate to considerable heterogeneity (Figure S1, Supplementary Materials).

As the severity of depression may also influence the effect of n-3 PUFAs and more severe depressions may result in greater effects of n-3 PUFAs [58], a subgroup analysis of studies including either individuals (a) with major depression/mild to moderate depression, (b) with no depression/mild to moderate depression, or (c) with any severity of depression was conducted. There was no statistically significant difference among subgroups (chi^2^ = 1.16, df = 2, *p* = 0.56, *I*^2^ = 0%) and a statistically significant beneficial effect was only seen in the subgroup of studies in participants with no depression/mild to moderate depression (SMD = −0.27, 95% CI: −0.53, −0.02). Heterogeneity was moderate to considerable in the three subgroups (Figure S2, Supplementary Materials).

Previous studies indicated that EPA may be more effective in improving depressive symptomology [9]. Therefore, we conducted a subgroup analysis in order to compare studies that supplied dosages of ≥1000 mg/day of EPA versus those with EPA <1000 mg/day. Subgroups were significantly different (*p* = 0.05, *I*^2^ = 74.4%, *n* = 3131). There was a statistically significant beneficial effect in the subgroup with lower EPA only: SMD = −0.82, 95% CI: −1.15, −0.14, *I*^2^ = 95%. Heterogeneity was moderate to considerable in the two subgroups (Figure S3, Supplementary Materials).

### 3.5. Sensitivity Analyses

Sensitivity analysis including only the nine studies with low risk of bias for which results could be pooled [24,25,26,39,47,48,56,59,60] seems to confirm the overall effect of n-3 PUFAs on depressive symptoms with an SMD of −0.33, 95% CI: −0.69, 0.03, albeit without reaching statistical significance. Heterogeneity was considerable with an *I*^2^ estimate of 85%. Similarly, including only studies with ITT analysis indicated no statistically significant beneficial effect of n-3 PUFAs (SMD = −0.22, 95% CI: -0.46, 0.01, *I*^2^ = 78%). When we excluded only studies with high risk of bias [61,62] from the analysis, there was still evidence for the lowering effect of n-3 PUFAs on depressive symptoms, similar to the overall result (SMD = −0.33, 95% CI: −0.56, −0.10, *I*^2^ = 87%). Leave-one-out sensitivity analyses demonstrated that the overall result was not influenced by any specific study included, but the pooled estimates were lowered by around one-third if any of the studies with strongest effects were excluded: SMD = −0.24, 95% CI: −0.43, −0.05 [26]; SMD = −0.22, 95% CI: −0.39, −0.05 [27] (Table S3, Supplementary Materials).

### 3.6. Descriptive Synthesis of Studies Not Included in the Meta-Analysis

Five studies could not be included in the pooled analysis because of missing outcome data. Two of those investigated the effects of n-3 PUFAs versus placebo in older participants with mild cognitive impairments [40,43] or physical function limitations [40]. While a large, high-quality study found no effect of a 3 year intervention with n-3 PUFA compared to placebo [40], in a small study which had a high risk of bias, GDS scores were improved in the EPA and DHA groups after 6 months of intervention [43]. Two studies investigated the effects of n-3 PUFA treatment in patients with cardiovascular diseases. One study included patients after acute myocardial infarction and compared the effects of n-3 PUFA supplementation of 1 g/day for 4 weeks versus standard therapy without placebo. The BDI score was significantly lower in the n-3 PUFA group at follow-up. Furthermore, after adjustment for age, sex, body mass index (BMI), and markers of disease severity, a beneficial effect of n-3 PUFA on the BDI score and a partial score of the Emotional State Questionnaire covering anger, disappointment and depression was observed [41]. In contrast, others found no effect of 1.9 g/day of n-3 PUFAs as compared to placebo on BDI-II and HRDS scores in patients with coronary artery disease [42]. In a small randomized placebo-controlled study in young adults with depressive symptoms, a 21 day treatment of 1.4 g/day of n-3 PUFAs resulted in a reduction in depressive symptomology [57]. In general, among these five studies, those with low risk of bias and higher sample size (total *n* = 1617) [40,42] found no effect of n-3 PUFA supplementation, whereas those with high risk of bias and lower sample size (total *n* = 123) [41,43,57] reported beneficial effects.

## 4. Discussion

Our meta-analysis of RCTs published between 2010 and 2020 suggests a modest beneficial effect of n-3 PUFA supplementation on depressive symptoms. The funnel plot, however, indicated potential presence of a reporting bias. Moreover, there was substantial heterogeneity between studies which was not explained by treatment duration, presence of comorbidity, or severity of depression, while EPA dosage may have contributed to heterogeneity. Among RCTs included in the meta-analysis, 25% were judged as poor quality, reflecting a high risk of bias.

A previously updated meta-analysis [63] based on a Cochrane review by Appleton et al. (2015) [23] investigated the effects of n-3 PUFAs on MDD in adults and found a small to modest benefit for depressive symptomology (SMD = −0.32, 95% CI: −0.52, −0.12) similar to our results. Further previous meta-analyses reported beneficial effects of n-3 PUFAs on depressive symptoms [18,64,65,66], whereas others did not find such an effect [17,21,67]. However, in one of the latter studies [21], some weaknesses including the selection of studies have been criticized [65,68].

In line with previous meta-analyses [21,66,67], our results do not suggest differences between longer or shorter treatment durations. This may be due to the fact that there are different biological mechanisms for n-3 PUFAs to influence depressive disorders, which operate on different time scales. Some of them can occur rapidly such as effects on cytokines in blood plasma, whereas other effects take longer, e.g., when n-3 PUFAs are incorporated into cell membranes. Given that the net DHA incorporation for the entire human brain is equal to only 3.8 ± 1.7 mg/day and that the half-life of DHA in the brain is about 2.5 years, short-term effects of n-3 PUFA seem to be based on mechanisms other than increases in n-3 PUFA concentration in brain membranes [69]. As persistent levels of EPA and DHA in blood plasma are established within 2 weeks [70], these mechanisms may include effects on cytokines with subsequent influence on neuropsychiatric functioning, as well as effects on the composition of newly formed neuronal membranes or on synaptic remodeling and neurogenesis [69]. Studies have indicated that blood fatty acid concentrations may serve as predictive biomarkers for the responsiveness to antidepressant treatment. Therefore, the monitoring of blood fatty acid levels should be considered [11].

Comorbidities may have a moderating impact on the effect of n-3 PUFA supplementation, possibly limiting beneficial effects of n-3 PUFAs only to non-comorbid patients [64]. However, our subgroup analysis did not indicate differences between subgroups by comorbidity status. This is in line with other meta-analyses [23,67].

In agreement with one previous meta-analysis [66], our results do not support differences between subgroups of less or more severe symptomology. A meta-analysis conducted in older participants including six studies indicated a beneficial effect of n-3 PUFAs in participants with mild to moderate depression but not in healthy participants [30]. Other meta-analyses found a larger effect when participants with more severe depression (MDD or moderate vs. mild; moderate vs. mild) were included [21,65].

Contrary to our results, several other meta-analyses reported stronger beneficial effects with higher EPA [65,66]. Our results, however, corroborate the findings of a recent meta-analysis [18], where a subgroup analysis indicated that studies with lower EPA dosage of ≤1 g/day resulted in beneficial effects, whereas, with higher dosages, no significant effect was detected.

We included studies since 2010 only and, therefore, our meta-analysis provides an update based on evidence of most recent trials in addition to the results of previous meta-analyses. In 2001, the CONSORT (Consolidated Standards of Reporting Trials) statement was established in order to improve the reporting of key methodological items of RCTs, which was updated in 2007 and 2010 as new concerns emerged regarding selective outcome reporting [71]. We expected that authors of newer RCTs would more often adhere to the CONSORT statement and checklist than those of older studies and would, therefore, be at a lower risk of bias by considering improved designs and reporting. Among the studies included in our review, the CONSORT checklist was shown in two publications [39,49] and the flow chart was presented in nine publications [24,25,26,27,49,51,56,59,72]. None of these studies were judged as being at high risk of bias. Overall, almost half of the studies included in our review were judged as being of good quality, and only five of 25 studies were judged as poor quality. Data from another five of 25 studies could not be included in the pooled analysis because of missing outcome data.

Limitations of our study include that we did not adjust for multiple tests and that our findings in subgroup analysis are based on subgroups which were not randomly assigned in the original RCTs, both of which might reduce the validity of our results. Additionally, the definition of no depression or mild to moderate depression may vary between the studies as it was adopted from the study authors’ assessment.

In contrast to previous systematic reviews and meta-analyses, our study included a broader range of trials. We allowed study populations of any age group, whereas several meta-analyses excluded studies in children and adolescents [17,18,23]. Contrary to others [23], we did not restrict the inclusion regarding severity of the disease. Moreover, studies in participants with comorbidities apart from psychotic or neurodegenerative diseases were not excluded as in a very recent meta-analysis [22], nor were studies with short treatment duration as in another one [17]. This seems to be a strength of our study, because, as discussed above, the beneficial effects of n-3 PUFAs can result from several different mechanisms and any of these can be expected to be effective independent of a specific study population.

As n-3 PUFA supplementation is a safe therapy and no adverse effects are to be expected, apart from mild gastrointestinal symptoms [73], n-3 PUFAs may provide a benefit if they are effective.

## 5. Conclusions

In summary, our results suggest a modest effect of n-3 PUFAs on depressive symptomology compared to placebo. However, the evidence is limited as 25% of studies included in the meta-analysis had a high risk of bias, and funnel plot inspection indicated that the result may be biased. Thus, according to the present systematic review and meta-analysis, n-3 PUFAs cannot generally be recommended as a treatment for depression. There is still a need for large, high-quality studies.

## Figures and Tables

**Table 1:** Characteristics of randomized controlled trials (RCTs) included in systematic review.

Lead Author, Publication Date	Country	Sample Size	Duration (Weeks)	Diet/Supplement (per Day)		Mean Age ± SD	Sex (%)		Health Status	Severity of Depression
				Intervention	Control		f	m		
Andrieu et al., 2017	France	1525	144 (36 months)	Group 1: capsule: 800 mg DHA 225 mg EPA + multidomain intervention	Group 3: placebo capsule: paraffin oil + multidomain intervention	75.3 ±4.4	64	36	Spontaneous memory complaints or limits in one instrumental activity of daily life or slow gait speed	Nondepressed and mild depression
				Group 2: capsule: 800 mg DHA 225 mg EPA	Group 4: placebo capsule: paraffin oil					
Antypa et al., 2011	Netherlands	71	4	Fish oil capsule: 1740 mg EPA 250 mg DHA	Placebo capsule: olive oil	24.65	81.65	18.35		Mild to moderate
Bot et al., 2010	Netherlands	25	12	Capsule: 1000 mg EPA	Placebo capsule: rapeseed oil + medium chain triglycerides	54.05	52	48	Diabetes mellitus 1 or 2	MDD
Carney et al., 2019	USA	144	10	Capsule: 2000 mg EPA sertraline (50 mg/day)	Placebo capsule sertraline (50 mg/day)	59.5	38.89	61.11	With or at risk of coronary heart disease	MDD
Chang et al., 2019	China	59	12	Capsule: 2000 mg EPA 1000 mg DHA	Placebo capsule: 300 mg soybean oil	61.5 ± 9	36	64	CVD	MDD
Gabbay et al., 2019	USA	48	10	Capsule: starting with 1200 mg, which was increased 600 mg every 2 weeks, up to a maximum of 3600 mg (2400 mg EPA, 1200 mg DHA)	Placebo capsule: 1:1 ratio of corn and soybean oils, consisting mainly n-6-PUFA (50%) and MUFA (25%)	16.05 ± 2.079	58.3	41.7		MDD
Gharekhani et al., 2014	Iran	54	16	Capsule: 1080 mg EPA 720 mg DHA	Placebo capsule: paraffin oil	57	44.44	55.56	Hemodialysis patients	Mild, moderate, severe
Giltay et al., 2011	Netherlands	4116	160	Group 1: margarine spread: 400 mg EPA + DHA 2 mg ALA	Group 4: placebo margarine: oleic acid	68.725	20.8	79.2	Myocardial infarct survivors	Mild, moderate, severe
				Group 2: margarine spread: 400 mg EPA + DHA						
				Group 3: margarine spread: 2 mg ALA						
Ginty et al., 2015	USA	21	3	Capsule: 1000 mg EPA 400 g DHA	Placebo capsule: corn oil	20.2	78	22		Mild to moderate
Haberka et al., 2013	Poland	52	4	Capsule: 46 5 mg EPA 375 mg DHA	Standard therapy, no placebo	58	13.46	86.54	Acute myocardial infarction	Minimal to moderate
Jahangard et al., 2018	Iran	50	12	Capsule: 1000 mg n3-PUFA sertraline (50–200 mg/day	Placebo capsule sertraline (50–200 mg/day)	42.46	68	32		MDD
Jiang et al., 2018	USA	108	12	Group 1: capsule: 2000 mg 2:1 EPA:DHA	Group 3: placebo capsule: corn oil	57.91	53.7	46.3	Chronic heart failure	MDD
				Group 2: capsule: 2000 mg EPA						
Khajehnasiri et al., 2012 *	Iran	136	15	Group 1: softgel: 360 mg EPA 240 mg DHA + capsule: 500 mg vit.C	Group 3: softgel placebo: paraffin oil + capsule: 500 mg vit.C	30.75	0	100		Mild to moderate
				Group 2: softgel: 360 mg EPA 240 mg DHA	Group 4: placebo softgel + placebo capsule					
Lespérance et al., 2011	Canada	432	8	Capsule: 1050 mg EPA 150 mg DHA	Placebo capsule: sunflower oil 2% fish oil	46	68.5	31.50	Only participants with specific comorbidities excluded	Major depressive episode
Mazereeuw et al., 2016	Canada	92	12	Capsule: 1200 mg EPA 600 mg DHA 100 mg other n-3 PUFA	Placebo capsule: 1:1 soybean/corn oil blend	61.7 ± 8.7	24	76	Coronary heart disease (in cardiac rehabilitation)	Nondepressed and minor to major depression
Mischoulon et al., 2015	USA	177	8	Group 1: capsule: 1060 mg EPA 274 mg DHA	Group 3: placebo capsule: 1000 mg soybean oil 50% LA (n-6-PUFA) 8% LA (n-3-PUFA)	45.8 ± 12.5	59.3	40.7		MDD
				Group 2: capsule: 450 mg DHA 90 mg EPA						
Mozaffari-Khosravi et al., 2013	Iran	62	12	Group 1: capsule: 1000 mg EPA	Placebo capsule: coconut oil	35.1 ± 1.2	61.3	38.7		Mild to moderate
				Group 2: capsule: 1000 mg DHA						
Ravi et al., 2016	Iran	100	8	Capsule: 720 mg EPA 480 mg DHA	Placebo capsule: olive oil	39.67	35	65	HIV positive	Moderate to severe depression
Rondanelli et al., 2010 ^†^	Italy	46	8	Fish oil capsule: 1670 mg EPA 830 g DHA/day	Placebo capsule: paraffin oil	83.95	100	0	Only participants with specific comorbidities excluded	MDD
Shinto et al., 2016	USA	31	12	Fish oil capsule: 1950 mg EPA 1350 mg DHA	Placebo capsule: soybean oil, 1% fish oil	51.3	18	82		MDD
Sinn et al., 2012	Australia	50	24	Group 1: fish oil capsule: 1670 mg EPA 160 mg DHA	Group 3: safflower oil placebo capsule:2200 mg LA (n-6 PUFA)	74.03	32	68	Self-reported memory loss, comorbidities, e.g., diabetes mellitus	Nondepressed and mild depression
				Group 2: fish oil capsule: 1550 mg DHA 400 mg EPA						
Tajalizadekhoob et al., 2011	Iran	66	24	Fish oil capsule: 180 mg EPA 120 mg DHA	Placebo capsule: coconut oil	69.685	69.70	30.30	Comorbidities, e.g., diabetes mellitus, hypertension, CVD, thyroid dysfunctions	Mild to moderate
Tayama et al., 2019	Japan	79	12	Capsule: 1064 mg EPA 558 mg DHA pysychoeducation	Placebo capsule: 705 mg rapseed oil 375 mg soybean oil 375 mg olive oil 45 mg fish oil psychoeducation	40.4	47.78	52.22	Only participants with specific comorbidities excluded	Mild to moderate
Trebaticka et al., 2020	Slovakia	58	12	Fish oil emulsion: 1000 mg EPA 750 mg DHA	Placebo emulsion: sunflower oil with 2467 mg n-6 LA	15.6 ±1.6	79.31	20.69	Only participants with specific comorbidities excluded	Depressive disorder with/without anxiety disorder
Watanabe et al., 2018	Japan	80	13	Group 1: capsule: 1200 mg EPA 600 mg DHA + stress management program	Group 3: placebo capsule: 47% rapeseed oil 25% soybean oil 25% olive oil 3% fish oil + stressmanagement program	30.1 ±8.4	100	0		Nondepressed or mild depression
				Group 2: capsule: 1200 mg EPA 600 mg DHA + psychoeducation	Group 4: placebo capsule: 47% rapeseed oil 25% soybean oil 25% olive oil 3% fish oil + psychoeducation					

ALA, alpha-linoleic acid; DHA, docosahexaenoic acid; CVD, cardiovascular disease; EPA, eicosapentanoic acid; HIV, human immunodeficiency virus; LA, linoleic acid; MDD, major depressive disorder; MUFA, monounsaturated fatty acids; n-3 PUFA, omega-3 polyunsaturated fatty acid; n-6 PUFA, omega-6 polyunsaturated fatty acid; SD, standard deviation; USA, United States of America; vit.C, vitamin C. * Results were also published in [44]. ^†^ Results were also published in [45,46].

**Table 2:** Summary of risk of bias assessment of the randomized controlled trials (RCTs) based on the Cochrane risk of bias tool *.

Lead Author, Year of Publication	Random Sequence Generation	Allocation Concealment	Selective Reporting	Blinding of Participants/Personnel	Blinding of Outcome Assessment	Incomplete Outcome	Other Bias	Study ^†^ Quality
Andrieu et al., 2017	+	+	+	+	+	+	+	Good
Antypa et al., 2011	+	+	+	+	+	+	+	Good
Bot et al., 2010	?	+	+	+	+	?	+	Fair
Carney et al., 2019	+	+	+	+	+	+	+	Good
Chang et al., 2019	?	?	+	?	?	+	+	Poor
Gabbay et al., 2019	?	+	+	+	+	+	+	Good
Gharekhani et al., 2014	+	?	+	−	?	+	+	Poor
Giltay et al., 2011	+	+	+	+	+	−	+	Fair
Ginty et al., 2015	+	+	−	+	+	−	+	Poor
Haberka et al., 2013	+	?	?	−	+	+	+	Poor
Jahangard et al., 2018	+	+	+	+	?	+	+	Good
Jiang et al., 2018	+	+	+	+	+	?	+	Good
Khajehnasiri et al., 2013	+	+	+	+	+	−	+	Fair
Lesperance et al., 2010	+	+	+	+	+	?	+	Good
Mazereeuw et al., 2016	+	+	+	+	+	+	+	Good
Mischoulon et al., 2015	+	+	+	+	?	?	+	Fair
Mozaffari-Khosravi et al., 2012	+	+	+	+	+	−	+	Fair
Ravi et al., 2016	+	+	+	+	+	−	+	Fair
Rondanelli et al., 2010	+	+	+	+	+	+	+	Good
Shinto et al., 2016	+	+	+	+	+	−	+	Fair
Sinn et al., 2012	+	?	+	+	+	−	+	Poor
Tajalizadekhoob et al., 2011	+	+	+	+	+	−	+	Fair
Tayama et al., 2018	+	+	+	+	+	−	+	Fair
Trebatická et al., 2020	+	+	+	+	+	+	+	Good
Watanabe et al., 2018	+	?	+	+	+	+	+	Good

* + = low risk of bias, − = high risk of bias, ? = unclear risk of bias. ^†^ Thresholds for converting the Cochrane risk of bias tool to AHRQ (Agency for Healthcare Research and Quality) standards (good, fair, and poor): good quality, all criteria met (i.e., low risk for each domain). Even if one criterion is not met, a study can be judged as good if the assessment was unlikely to have biased the outcome and there is no known important limitation that could invalidate the results; fair quality, one criterion not met (i.e., high risk of bias for one domain) or two criteria unclear, and the assessment that this was unlikely to have biased the outcome, and there is no known important limitation that could invalidate the results; poor quality, one criterion not met (i.e., high risk of bias for one domain) or two criteria unclear, and the assessment that this was likely to have biased the outcome, and there are important limitations that could invalidate the results. Two or more criteria listed as high or unclear risk of bias.

## Data Availability

Not applicable.

## Supplementary Materials

The following are available online at https://www.mdpi.com/2072-6643/13/4/1070/s1: Figure S1: Effect of n-3 PUFA on depressive symptoms by subgroup of studies including participants with comorbidities, without comorbidities or with and without comorbidities, Figure S2: Effect of n-3 PUFA on depressive symptoms by subgroup of studies including participants with major depression only, with mild to moderate and severe depression, without depression and mild to moderate depression, Figure S3: Effect of n-3 PUFA on depressive symptoms by subgroup of studies with high or low EPA dosage (≥ versus <1000 mg/d), Table S1: Search strategy used in current review, Table S2: Baseline and end-study estimates and/or mean changes of trials included in the systematic review, Table S3: Leave-one-out sensitivity analyses.
