# Supplementary material for: Effects of n-3 Polyunsaturated Fatty Acid Supplementation in the Prevention and Treatment of Depressive Disorders—A Systematic Review and Meta-Analysis
Source: Nutrients. 2021 Mar 25;13(4):1070. doi: 10.3390/nu13041070 (PMC8064470; doi:10.3390/nu13041070)
Supplement: Supplementary file 1 [file nutrients-13-01070-s001.pdf]

Supplementary Material for the article

Wolters et al. Effects of n-3 polyunsaturated fatty acid supplementation in the prevention and treatment of depressive disorders – a systematic review and meta-analysis

Supplementary Table S1. Search strategy used in current review. All searches were conducted on January 1, 2020.

**MEDLINE via Ovid**<br>1. (depression* or "depressive disorder*" or dysthymia or blues or dejection or despondency or disconsolate or dumps or gloom or joylessness or mournfulness or sadness or sorrowfulness or unhappiness).ti,ab.<br>2. exp depression/ or exp depressive disorder/ or exp depressive disorder, major/ or exp dysthymia/ or exp sadness/<br>3. 1 or 2<br>4. ("omega 3 fatty acid*" or "omega-3 fatty acid*" or "n 3 fatty acid*" or "n-3 fatty acid*" or "w 3 fatty acid*" or "w-3 fatty acid*" or "ω 3 fatty acid*" or "ω-3 fatty acid*" or "eicosapentaenoic acid*" or EPA or "alpha linolenic acid" or ALA or "docosahexaenoic acid*" or DHA or "fish oil" or "cod liver oil" or PUFA or "polyunsaturated fatty acid*").ti,ab.<br>5. exp acids, omega 3 fatty/ or exp fatty acids, unsaturated/ or exp eicosapentaenoic acid/ or exp alpha linolenic acid/ or exp docosahexaenoic acid/ or exp fish oils/ or exp cod liver oil/<br>6. 4 or 5<br>7. 3 and 6<br>8. limit 7 to (humans and yr="2010 - 2020")<br>9. limit 8 to randomized controlled trial
**PSYCINFO via Ovid**<br>1. (depression* or "depressive disorder*" or dysthymia or blues or dejection or despondency or disconsolate or dumps or gloom or joylessness or mournfulness or sadness or sorrowfulness or unhappiness).ti,ab.<br>2. exp "Major Depression"/ or exp "Depression (Emotion)"/ or exp "Dysthymic Disorder"/ or exp "Sadness"/<br>3. 1 or 2<br>4. ("omega 3 fatty acid*" or "omega-3 fatty acid*OR n 3 fatty acid*" or "n-3 fatty acid*" or "w 3 fatty acid*" or "w-3 fatty acid*" or "ω 3 fatty acid*" or "ω-3 fatty acid*" or "eicosapentaenoic acid*" or EPA or "alpha linolenic acid" or ALA or "docosahexaenoic acid*" or DHA or "fish oil" or "cod liver oil" or PUFA or "polyunsaturated fatty acid*").ti,ab.<br>5. exp "Fatty Acids"/<br>6. 4 or 5<br>7. 3 and 6<br>8. limit 7 to (human and "0300 clinical trial" and yr="2010 - 2020“)
**CENTRAL via the Cochrane Library**<br>ID Search Hits<br>#1 MeSH descriptor: [Depression] explode all trees 10762<br>#2 MeSH descriptor: [Depressive Disorder] explode all trees 11061<br>#3 MeSH descriptor: [Dysthymic Disorder] explode all trees 168<br>#4 MeSH descriptor: [Sadness] explode all trees 3<br>#5 (depression* OR "depressive disorder*" OR dysthymia OR blues OR dejection OR despondency OR disconsolate OR dumps OR gloom OR joylessness OR mournfulness OR sadness OR sorrowfulness OR unhappiness):ti,ab 63158<br>#6 MeSH descriptor: [Fatty Acids, Omega-3] explode all trees 2933<br>#7 MeSH descriptor: [Eicosapentaenoic Acid] explode all trees 991

#8 MeSH descriptor: [Docosahexaenoic Acids] explode all trees 1125
#9 MeSH descriptor: [alpha-Linolenic Acid] explode all trees 227
#10 MeSH descriptor: [Fish Oils] explode all trees 3252
#11 MeSH descriptor: [Cod Liver Oil] explode all trees 34
#12 MeSH descriptor: [Fatty Acids, Unsaturated] explode all trees 12050
#13 ("omega 3 fatty acid*" OR "omega-3 fatty acid*" OR "n 3 fatty acid*" OR "n-3 fatty acid*" OR "w 3 fatty acid*" OR "w-3 fatty acid*" OR "ω 3 fatty acid*" OR "ω-3 fatty acid*" OR "eicosapentaenoic acid*" OR EPA OR "alpha linolenic acid" OR ALA OR "docosahexaenoic acid*" OR DHA OR "fish oil" OR "cod liver oil" OR PUFA OR "polyunsaturated fatty acid*"):ti,ab 8798
#14 ((#1 OR #2 OR #3 OR #4) OR #5) AND ((#6 OR #7 OR #8 OR #9 OR #10 OR #11 OR #12) OR #13) with Publication Year from 2010 to 2020, in Trials 313

Supplementary Table S2: Baseline and end-study estimates and/or mean changes of trials included in the systematic review (Studies in lines marked in light grey were not included in the meta-analysis)

Lead author, publication date	Intervention (n-3 PUFA dosage/d)	Outcome assessment score	Intervention group			Placebo group		
			Sample size	Baseline mean ± SD	End study mean ± SD and/or mean change ± SD	Sample size	Baseline mean ± SD	End study mean ± SD
**Andrieu et al. 2017[1]**	Group 1: capsule: 800mg DHA 225mg EPA + multidomain intervention	Geriatric Depression Scale (GDS)	374	3.17 ±2.47	mean change: -0.280 ±2.659	390	3.2 ±2.58	mean change: -0.126 ±1.265
	Group 2: capsule: 800mg DHA 225mg EPA		381	3.27 ±2.67	mean change: -0.444 ±2.694	380	3.21 ±2.7	mean change: -0.295 ±2.660
**Antypa et al. 2011**	fish oil capsule: 1740mg EPA 250mg DHA	Beck Depression Inventory II (BDI-II)	36	5.7±5.0	6.6±7.3	35	7.7±5.9	6.5±6.4
**Bot et al. 2010**	capsule: 1000mg E-EPA	Montgomery Asberg Depression Rating Scale (MADRS)	12	26.3 ±8.2	14.0 ±6.9	12	26.4 ±8.7	11.6 ±9.1
**Carney et al. 2019 (pers. commun.)**	capsule: 2000mg EPA + 50mg Sertralin	Beck Depression Inventory II (BDI-II)	71	29.9±9.0	11.0 ±9.0; mean change: -18.9±12.9	73	29.1 ±8.8	9.1±7.7; mean change: -9.7±9.7

		Hamilton Depression Rating Scale (HDRS)		17.4 ±5.4	7.1±7; mean change: -11.3±8.7		17.0 ±5.0	6.2±5.5; mean change: -11.3±6.9
		Patient Health Questionaire (PHQ-9)		15.9 ±4.6	5.4±5.2; mean change: -11.2±8.1		15.4 ±4.1	4.9±4.7; mean change: -10.8±5.4
**Chang et al. 2019[2] (pers. commun.)**	capsule: 2000mg EPA 1000mg DHA	Hamilton Depression Rating Scale (HDRS)	30	19 ±4.21	13.90 ±4.44 mean change: -5.10 ±4.05	29	19.17 ±3.58	15.07± 3.90 mean change: -4.10 ±3.42
		Beck Depression Inventory (BDI)		17.67 ±9.08	14.73 ± 10.75 mean change: -3.07 ±5.44		17.72 ±6.31	14.17± 7.76 mean change: -2.97 ±7.47
**Gabbay et al. 2019**	capsule: starting with 1200mg, which was increased 600mg every 2 weeks, up to a maximum of 3600mg (2400mg EPA, 1200mg DHA)	Children's Depression Rating Scale-Revised (CDRS-R)	21	49.5 ±8.20	36.5 ±10.01	27	50.2 ± 8.91	35.2 ±10.57
		Beck Depression Inventory-II (BDI-II)		24.2 ±13.70	16.9 ±13.21		22.4 ±12.65	14.8 ±11.97
**Gharekhani et al. 2014 (pers. commun.)**	capsules: 1080mg EPA 720mg DHA	Beck Depression Inventory (BDI)	27		14.56 ±6.8 mean change: -10.68 ±8.28	27		20.4 ±6.69 mean change: -0.98 ±8.71

	Group 1: Magarine spread:<br>400mg EPA + DHA<br>2mg ALA/d		1021		2.1060 ± 2.4106 mean change (N=170): 0.2342 ±2.1352			
	Group 2: Magarine spread:<br>400mg EPA + DHA		1015		2.0161± 2.3252 mean change (N=146): 0.3064 ±1.7676			
**Giltay et al. 2011 (pers. commun.)**	Group 3: Magarine spread:<br>2mg ALA	Geriatric Depression Scale (GDS)	1034		2.0466 ± 2.3597 mean change (N=162): 0.0770 ±2.2588	1046		2.1631 ± 2.4591 mean change (N=173): -0.0127 ±1.8416
**Ginty et al. 2015**	capsule:<br>1000mg EPA<br>400mg DHA	Beck Depression Inventory (BDI)	12	15.58 ±5.21	NA	9	15.89 ±5.46	NA
**Haberka et al. 2013**	capsule:<br>465mg EPA<br>375mg DHA	Beck Depression Iventory (BDI)	26	11.1 ±6.8	9.3	26	11.7 ±6.8	11.3

		Beck Depression Inventory (BDI)		36.36 ±6.0	7.16 ±1.70		35.44 ±6.24	15.44 ±3.62
**Jahangard et al. 2018**	capsule:<br>1000mg n3-PUFA<br>+ 50-200mg Sertraline	Montgomery Asberg Depression Rating Scale (MADRS)	25	37.2 ±7.57	6 ±2.38	25	32.60 ±7.53	17.20 ±3.52
	Group 1: capsule:<br>2000mg 2:1 EPA:DHA	Hamilton Depression Scale (HDRS)	Group 1: 36	Group 1: 22.22 ±3.78<br>Group 2: 22.67 ±4.24	15.1 ±5.4<br>15.7 ±5.4		23.56 ±4.53	14.9 ±5.4
**Jiang et al. 2018[2]**	Group 2: capsule:<br>2000mg EPA	Beck Depression Inventory II (BDI-II)	Group 2: 36	Group 1: 19 ±7.58<br>Group 2: 20 ±8.45	13.3 ±7.8<br>13.6 ±7.8	36	21.53 ±8.47	14.3 ±7.8
	Group 1: softgel:<br>360 mg EPA<br>240mg DHA<br>+ capsule:<br>500mg Vit.C		34	14.242 ±3.71	11.18±5.31 mean change: -3.061 ±6.83	34	14.133 ±4.37	10.90±5.90 mean change: -3.233 ±7.08
**Khajehnasiri et al. 2012[2]**	Group 2: softgel:<br>360 mg EPA<br>240mg DHA	Beck Depression Inventory (BDI)	34	13.903 ±4.63	7.61±5.16 mean change: -6.29 ±6.82	34	14.090 ±3.42	11.75±5.55 mean change: -2.34 ±5.88

		Inventory of Depressive Symptomatolgy (IDS-SR)		43.8 ±8.75	30.93 ±12.12[3]		43.3 ±8.88	32.26 ±12.16[3]
**Lespérance et al. 2011 (pers. commun.)**	capsule: 1050mg EPA 150mg DHA	Montgomery Asberg Depression Rating Scale (MADRS)	218		17.9 ±8.9[4]	214		18.8 ±8.9[4]
		Hamilton Depression Rating Scale (HDRS)		6.5 ±6.3	mean change: -1.1 ±4.6		7.4 ±5.6	NA
**Mazereeuw et al. 2016**	capsule: 1200mg EPA 600mg DHA 100mg other n-3 PUFA	Beck Depression Inventory II (BDI-II)	45	12.2 ±11.5	NA	47	12.4 ±10.5	NA
		Hamilton Depression Rating Scale (HDRS)		Group 1: 19.3 ±3.8	8.96 (6.9)[4] mean change: -10.34±4.8025			
				Group 2: 19.8 ±3.2	10.54 (6.9)[4] mean change: -9.26±4.7218		19.2 ±3.1	group 1 (N30), group 2 (N29): 9.71 (6.4)[4] mean change: -9.49±4.6855
	Group 1: capsule: 1060mg EPA 274mg DHA	Clinical Global Impression (CGI-S)		Group 1: 4.2 ±0.6	mean change: -1.46 ±0.8521			
			Group 1: 60	Group 2: 4.2 ±0.7	mean change: -1.33±0.8377		4.0 ±0.6	mean change: -1.41±0.8449
**Mischoulon et al. 2015[2,3] (pers. commun.)**	Group 2: capsule: 900mg DHA 180mg EPA	Quick Inventory of Depressive	Group 2:	Group 1: 12.9 ±3.9	mean change: -5.01±3.6406	59	13.5 ±3.8	mean change: -5.54±3.6101

		Symptomatology (QIDS-SR)	58	Group 2: 13.3 ±4.5	mean change: -4.79±3.5794			
Mozaffari-Khosravi et al. 2013[2]	Group 1: capsule: 1000mg EPA	Hamilton Depression Rating Scale (HDRS)	21	15.9 ±2	10.3 ± 3.208	21	15.5 ±2.3	13.7 ±2.750
	Group 2: capsule: 1000mg DHA		20	15.7 ±2.4	13.7 ±2.683			
Ravi et al. 2016	capsule: 720mg EPA 480mg DHA	Beck Depression Inventory II (BDI-II)	50	28.42 ±8.51	14.30 ±5.78	50	27.20 ±5.86	26.70 ±5.95
		Depression subscale of the Hospital Anxiety and Depression scale (HADS-D)		13.04 ±2.51	7.54 ±1.99		12.98 ±2.66	12.68 ±2.64
		Patient Health Questionaire (PHQ-9)		16.16 ±4.03	7.8 ±2.61		15.42 ±4.20	15.14 ±4.08
Rondanelli et al. 2010	fish oil capsule: 1670mg EPA 830mg DHA	Geriatric Depression Scale (GDS)	22	17.1 ±3.6	12.6 ±4.3	24	16.7 ±4.3	15.9 ±5.4
Shinto et al. 2016 (pers. commun.)	fish oil capsule: 1950mg EPA 1350mg DHA	Montgomery Asberg Depression Rating Scale (MADRS)	15	18.4 ±5.3	10.9 ±7.4 mean change: -6.9 ±8.3	16	19.1 ±4	9.0 ±5.2 mean change: -9.7 ±6.0
		Beck Depression Inventory (BDI)		20.1 ±8	10.6 ±5.8 mean change: -8.2 ±6.5		19.6 ±5.7	8.9 ±6.6 mean change: -11.1 ±5.8

Sinn et al. 2012	Group 1: fish oil capsule: 1670mg EPA 160mg DHA	Geriatric Depression Scale (GDS)	17	4.40 ±2.92	NA	15	3.15 ±2.08	NA
	Group 2: fish oil capsule: 1550mg DHA 400mg EPA		18	3.19 ±3.17	NA			
**Tajalizadekhoob et al. 2011**	fish oil capsule: 180mg EPA 120mg DHA	Geriatric Depression Scale (GDS)	33	7.24 ±1.95	6.00 ±2.92	33	7.21 ±1.83	6.91 ±3.98
**Tayama et al. 2019**	capsule: 1064mg EPA 558mg DHA + psychoeducation	Beck Depression Inventory II (BDI-II)	47	12.3 ±6.1	8.4 ±5.1	43	12.2 ±7.7	8.0 ±4.5
**Trebaticka et al. 2020 (pers. commun.)**	fish oil emulsion: 1000mg EPA 750mg DHA	Children's Depression Inventory (CDI)	29	27.8 ±9.0	21.34±11.7; mean change:--7.6±7.1	29	24.9 ±8.5	22.24 ±10.1; mean change:-2.9±7.8
**Watanabe et al. 2018[1]**	Group 1: capsule: 1200mg EPA 600mg DHA + stressmanagement program	Hospital Anxiety and Depression scale (HADS)	40	Group 1 and 2: 7.4 ±4.8	Week 13: 6.40 ±3.872 Week 26: 6.32 ±3.856 Week 52: 5.85 ±3.872	40	7.11 ± 4.55	Week 13: 7.60±3.985 Week 26: 6.81±4.001 Week 52: 8.32±4,050
		Hospital Anxiety and Depression scale (HADS), depression score		Group 1 and 2: 3.3 ±2.8	Week 13: 2.84 ±2.194 Week 26: 2.69 ±2.194 Week 52: 2.36 ±2.194		3.1 ±2.5	Week 13: 3.54 ±2.259 Week 26: 3.05 ±2.259 Week 52: 3.80 ±2.291

	Group 2: capsule: 1200mg EPA 600mg DHA + psychoeducation	Patient Health Questionaire (PHQ-9)		Group 1 and 2: 5.1 ±3.4	Week 13: 5.42 ±2.953 Week 26: 5.39 ±2.969 Week 52: 5.42 ±2.953		4.7 ±3.4	Week 13: 5.83 ±3.388 Week 26: 5.31 ±3.082 Week 52: 4.79 ±3.114

[1]To calculate the SD from 95% CI we used the following formula: SD= √N *(upper limit-lower limit)/3.92

[2]To calculate the SD from standard error we used the following formula: SD = √N ∗ SE

[3]For changes: Least squares means with adjustments are given

[4]Data taken from Appleton et al. 2015

Abbreviations: ALA, alpha-linoleic acid; DHA, docosahexaenoic acid; EPA, eicosapentanoic acid; ITT, intention to treat; MDD, major depressive disorder; NA, not available; pers. commun., personal communication; SD, standard deviation

Supplementary Table S3. Leave-one-out sensitivity analyses

Study	Standardized mean difference, given named study is omitted	95% Confidence interval	I^2 estimate
Antypa 2011	-0.36	-0.58, -0.13	87%
Bot 2010	-0.36	-0.58, -0.14	87%
Carney 2019	-0.37	-0.60, -0.14	87%
Chang 2019	-0.36	-0.58, -0.13	87%
Gabbay 2019	-0.36	-0.58, -0.14	87%
Gharekhani 2014	-0.31	-0.53, -0.09	87%
Giltay 2011	-0.37	-0.64, -0.09	86%
Jahangard 2018	-0.24	-0.43, -0.05	81%
Jiang 2018	-0.35	-0.58, -0.12	87%
Khajehnasiri 2012	-0.31	-0.53, -0.09	87%
Lespérance 2011	-0.36	-0.61, -0.11	87%
Mischoulon 2015	-0.36	-0.59, -0.13	87%
Mozzafari-Khosravi 2013	-0.33	-0.55, -0.10	87%
Ravi 2016	-0.22	-0.39, -0.05	77%
Rondanelli 2010	-0.32	-0.54, -0.10	87%
Shinto 2016	-0.36	-0.58, -0.14	87%
Tajalizadekhoob 2011	-0.34	-0.57, -0.11	87%
Tayama 2019	-0.36	-0.59, -0.13	87%
Trebatickà 2020	-0.35	-0.58, -0.12	87%
Watanabe 2018	-0.34	-0.56, -0.11	87%

Study or Subgroup	n-3 PUFA Mean	n-3 PUFA SD	n-3 PUFA Total	Placebo Mean	Placebo SD	Placebo Total	Weight	Std. Mean Difference IV, Random, 95% CI
**1.5.1 with comorbidities**								
Bot 2010	14	6.9	12	11.6	9.1	12	3.5%	0.29 [-0.52, 1.09]
Chang 2019	14.73	10.75	30	14.17	7.76	29	4.9%	0.06 [-0.45, 0.57]
Gharekhani 2014	14.56	6.8	27	20.4	6.69	27	4.6%	-0.85 [-1.41, -0.29]
Giltay 2011	2.0562	2.3649	3038	2.1631	2.4591	1030	6.7%	-0.04 [-0.12, 0.03]
Jiang 2018	13.45	7.7463	72	14.3	7.8	36	5.5%	-0.11 [-0.51, 0.29]
Ravi 2016	14.3	5.78	50	26.7	5.95	50	5.0%	-2.10 [-2.59, -1.61]
Shinto 2016	10.6	5.8	15	8.9	6.6	16	3.9%	0.27 [-0.44, 0.97]
**Subtotal (95% CI)**			**3244**			**1200**	**34.0%**	**-0.37 [-0.91, 0.17]**
Heterogeneity: Tau² = 0.45; Chi² = 74.97, df = 6 (P < 0.00001); I² = 92%								
Test for overall effect: Z = 1.36 (P = 0.17)								
**1.5.2 with/without comorbidities**								
Carney 2019	11	9	71	9.1	7.7	73	5.8%	0.23 [-0.10, 0.55]
Lespérance 2011	17.9	8.9	218	18.8	8.9	214	6.4%	-0.10 [-0.29, 0.09]
Rondanelli 2010	12.6	4.3	22	15.9	5.4	24	4.5%	-0.66 [-1.26, -0.07]
Tajalizadekhoob 2011	6	2.92	33	6.91	3.98	33	5.0%	-0.26 [-0.74, 0.23]
Tayama 2019	8.4	5.1	47	8	4.5	43	5.4%	0.08 [-0.33, 0.50]
Watanabe 2018	2.84	2.194	40	3.54	2.259	40	5.2%	-0.31 [-0.75, 0.13]
**Subtotal (95% CI)**			**431**			**427**	**32.3%**	**-0.11 [-0.32, 0.10]**
Heterogeneity: Tau² = 0.03; Chi² = 9.21, df = 5 (P = 0.10); I² = 46%								
Test for overall effect: Z = 1.04 (P = 0.30)								
**1.5.3 without comorbidities**								
Antypa 2011	6.6	7.3	36	6.5	6.4	35	5.1%	0.01 [-0.45, 0.48]
Gabbay 2019	16.9	13.21	21	14.8	11.97	27	4.6%	0.16 [-0.41, 0.74]
Jahangard 2018	7.16	1.7	25	15.44	3.62	25	3.5%	-2.88 [-3.69, -2.07]
Khajehnasiri 2012	7.61	5.16	34	11.75	5.55	34	5.0%	-0.76 [-1.26, -0.27]
Mischoulon 2015	9.7366	6.9161	118	9.71	6.4	59	5.9%	0.00 [-0.31, 0.32]
Mozaffari-Khosravi 2013	12.0415	3.3835	41	13.7	2.75	21	4.8%	-0.51 [-1.05, 0.02]
Trebatickà 2020	21.34	11.7	29	22.24	10.1	29	4.9%	-0.08 [-0.60, 0.43]
**Subtotal (95% CI)**			**304**			**230**	**33.7%**	**-0.52 [-1.07, 0.03]**
Heterogeneity: Tau² = 0.48; Chi² = 51.81, df = 6 (P < 0.00001); I² = 88%								
Test for overall effect: Z = 1.86 (P = 0.06)								
**Total (95% CI)**			**3979**			**1857**	**100.0%**	**-0.34 [-0.55, -0.12]**
Heterogeneity: Tau² = 0.18; Chi² = 140.42, df = 19 (P < 0.00001); I² = 86%								
Test for overall effect: Z = 3.03 (P = 0.002)								
Test for subgroup differences: Chi² = 2.42, df = 2 (P = 0.30), I² = 17.3%								

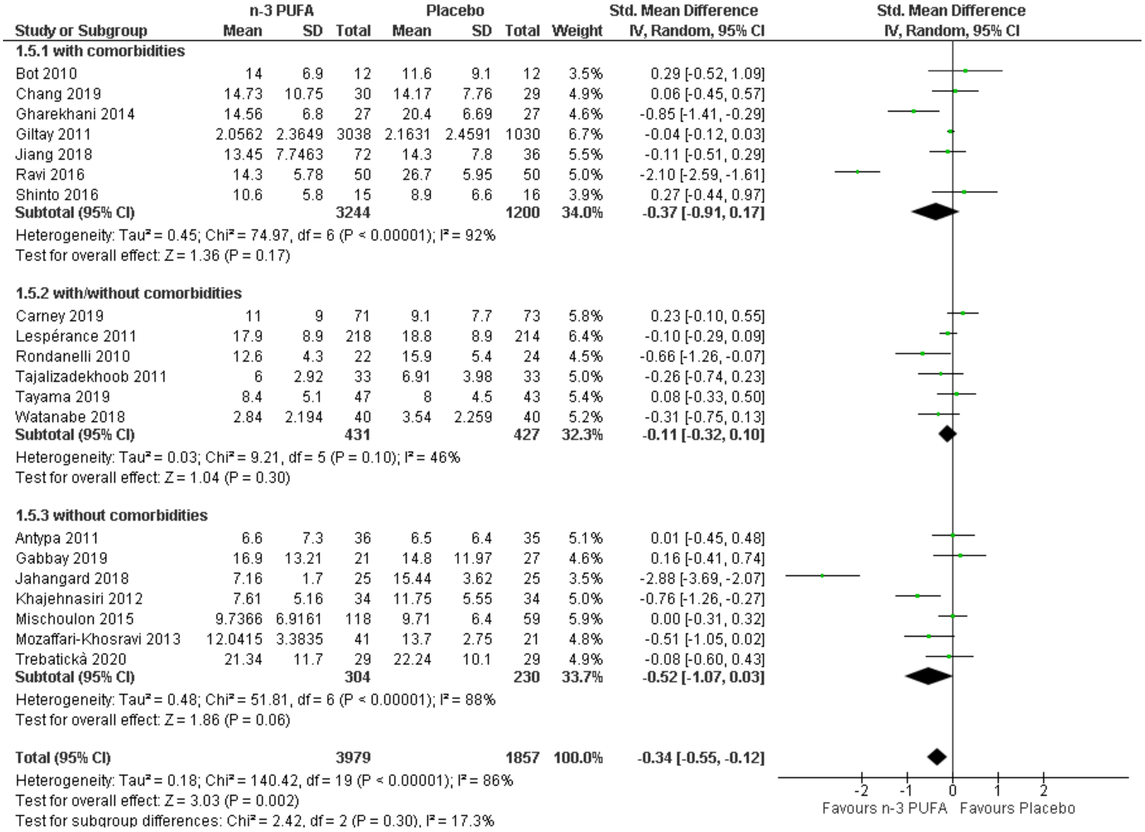

Supplementary Figure S1: Effect of n-3 PUFA on depressive symptoms by subgroup of studies including participants with comorbidities, without comorbidities or with and without comorbidities

Study or Subgroup	n-3 PUFA Mean	n-3 PUFA SD	n-3 PUFA Total	Placebo Mean	Placebo SD	Placebo Total	Weight	Std. Mean Difference IV, Random, 95% CI
**1.6.1 Major depressive disorder**								
Bot 2010	14	6.9	12	11.6	9.1	12	3.5%	0.29 [-0.52, 1.09]
Carney 2019	11	9	71	9.1	7.7	73	5.8%	0.23 [-0.10, 0.55]
Chang 2019	14.73	10.75	30	14.17	7.76	29	4.9%	0.06 [-0.45, 0.57]
Gabbay 2019	16.9	13.21	21	14.8	11.97	27	4.6%	0.16 [-0.41, 0.74]
Jahangard 2018	7.16	1.7	25	15.44	3.62	25	3.5%	-2.88 [-3.69, -2.07]
Jiang 2018	13.45	7.7463	72	14.3	7.8	36	5.5%	-0.11 [-0.51, 0.29]
Lespérance 2011	17.9	8.9	218	18.8	8.9	214	6.4%	-0.10 [-0.29, 0.09]
Mischoulon 2015	9.7366	6.9161	118	9.71	6.4	59	5.9%	0.00 [-0.31, 0.32]
Rondanelli 2010	12.6	4.3	22	15.9	5.4	24	4.5%	-0.66 [-1.26, -0.07]
Shinto 2016	10.6	5.8	15	8.9	6.6	16	3.9%	0.27 [-0.44, 0.97]
**Subtotal (95% CI)**			**604**			**515**	**48.3%**	**-0.21 [-0.55, 0.13]**
Heterogeneity: Tau² = 0.23; Chi² = 56.18, df = 9 (P < 0.00001); I² = 84%								
Test for overall effect: Z = 1.22 (P = 0.22)								
**1.6.2 Mild/moderate/severe depression**								
Gharekhani 2014	14.56	6.8	27	20.4	6.69	27	4.6%	-0.85 [-1.41, -0.29]
Giltay 2011	2.0562	2.3649	3038	2.1631	2.4591	1030	6.7%	-0.04 [-0.12, 0.03]
Ravi 2016	14.3	5.78	50	26.7	5.95	50	5.0%	-2.10 [-2.59, -1.61]
Trebaticką 2020	21.34	11.7	29	22.24	10.1	29	4.9%	-0.08 [-0.60, 0.43]
**Subtotal (95% CI)**			**3144**			**1136**	**21.1%**	**-0.76 [-1.69, 0.17]**
Heterogeneity: Tau² = 0.85; Chi² = 72.77, df = 3 (P < 0.00001); I² = 96%								
Test for overall effect: Z = 1.60 (P = 0.11)								
**1.6.3 No/mild/moderate depression**								
Antypa 2011	6.6	7.3	36	6.5	6.4	35	5.1%	0.01 [-0.45, 0.48]
Khajehnasiri 2012	7.61	5.16	34	11.75	5.55	34	5.0%	-0.76 [-1.26, -0.27]
Mozaffari-Khosravi 2013	12.0415	3.3835	41	13.7	2.75	21	4.8%	-0.51 [-1.05, 0.02]
Tajalizadekhoob 2011	6	2.92	33	6.91	3.98	33	5.0%	-0.26 [-0.74, 0.23]
Tayama 2019	8.4	5.1	47	8	4.5	43	5.4%	0.08 [-0.33, 0.50]
Watanabe 2018	2.84	2.194	40	3.54	2.259	40	5.2%	-0.31 [-0.75, 0.13]
**Subtotal (95% CI)**			**231**			**206**	**30.5%**	**-0.27 [-0.53, -0.02]**
Heterogeneity: Tau² = 0.04; Chi² = 8.89, df = 5 (P = 0.11); I² = 44%								
Test for overall effect: Z = 2.09 (P = 0.04)								
**Total (95% CI)**			**3979**			**1857**	**100.0%**	**-0.34 [-0.55, -0.12]**
Heterogeneity: Tau² = 0.18; Chi² = 140.42, df = 19 (P < 0.00001); I² = 86%								
Test for overall effect: Z = 3.03 (P = 0.002)								
Test for subgroup differences: Chi² = 1.16, df = 2 (P = 0.56), I² = 0%								

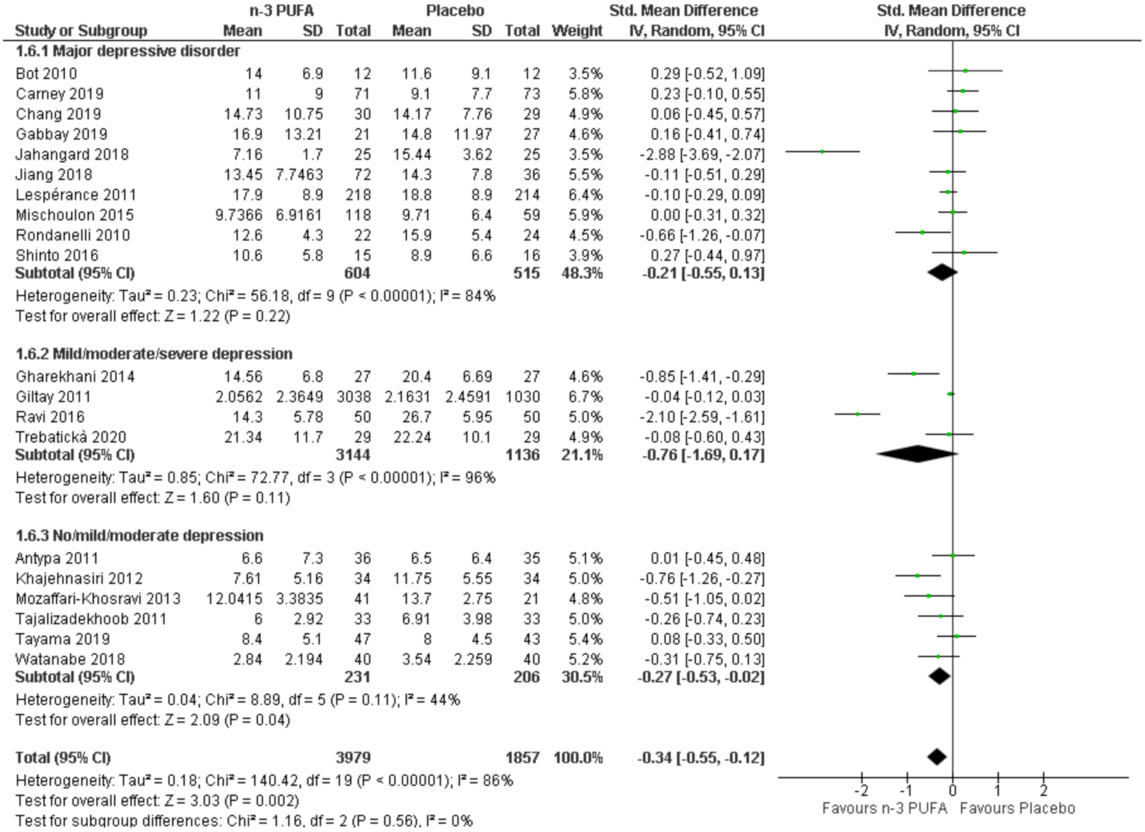

Supplementary Figure S2: Effect of n-3 PUFA on depressive symptoms by subgroup of studies including participants with major depression only, with mild to moderate and severe depression, without depression and mild to moderate depression.

Study or Subgroup	n-3 PUFA Mean	n-3 PUFA SD	n-3 PUFA Total	Placebo Mean	Placebo SD	Placebo Total	Weight	Std. Mean Difference IV, Random, 95% CI
**1.1.1 EPA ≥1000 mg**								
Antypa 2011	6.6	7.3	36	6.5	6.4	35	4.6%	0.01 [-0.45, 0.48]
Bot 2010	14	6.9	12	11.6	9.1	12	3.3%	0.29 [-0.52, 1.09]
Carney 2019	11	9	71	9.1	7.7	73	5.1%	0.23 [-0.10, 0.55]
Chang 2019	14.73	10.75	30	14.17	7.76	29	4.4%	0.06 [-0.45, 0.57]
Gabbay 2019	16.9	13.21	21	14.8	11.97	27	4.2%	0.16 [-0.41, 0.74]
Gharekhani 2014	14.56	6.8	27	20.4	6.69	27	4.2%	-0.85 [-1.41, -0.29]
Jiang 2018 (EPA only)	13.6	7.8	36	14.3	7.8	18	4.2%	-0.09 [-0.65, 0.48]
Jiang 2018 (EPA+DHA)	13.3	7.8	36	14.3	7.8	18	4.2%	-0.13 [-0.69, 0.44]
Lespérance 2011	17.9	8.9	218	18.8	8.9	214	5.6%	-0.10 [-0.29, 0.09]
Mischoulon 2015 (EPA)	8.96	6.9	60	9.71	6.4	30	4.7%	-0.11 [-0.55, 0.33]
Mozaffari-Khosravi 2013 (EPA)	10.3	3.208	21	13.7	2.75	11	3.3%	-1.08 [-1.86, -0.30]
Rondanelli 2010	12.6	4.3	22	15.9	5.4	24	4.1%	-0.66 [-1.26, -0.07]
Shinto 2016	10.6	5.8	15	8.9	6.6	16	3.6%	0.27 [-0.44, 0.97]
Tayama 2019	8.4	5.1	47	8	4.5	43	4.8%	0.08 [-0.33, 0.50]
Trebaticka 2020	21.34	11.7	29	22.24	10.1	29	4.4%	-0.08 [-0.60, 0.43]
Watanabe 2018	2.84	2.194	40	3.54	2.259	40	4.7%	-0.31 [-0.75, 0.13]
**Subtotal (95% CI)**			**721**			**646**	**69.4%**	**-0.11 [-0.27, 0.04]**
Heterogeneity: Tau² = 0.04; Chi² = 25.20, df = 15 (P = 0.05); I² = 40%								
Test for overall effect: Z = 1.42 (P = 0.16)								
**1.1.2 EPA < 1000 mg**								
Giltay 2011 (EPA+DHA)	2.0161	2.3252	1015	2.1631	2.4591	348	5.7%	-0.06 [-0.18, 0.06]
Jahangard 2018	7.16	1.7	25	15.44	3.62	25	3.2%	-2.88 [-3.69, -2.07]
Khajehnasiri 2012	7.61	5.16	34	11.75	5.55	34	4.5%	-0.76 [-1.26, -0.27]
Mischoulon 2015 (DHA)	10.54	6.9	58	9.71	6.4	29	4.7%	0.12 [-0.32, 0.57]
Mozaffari-Khosravi 2013 (DHA)	13.7	2.683	20	13.7	2.75	10	3.4%	0.00 [-0.76, 0.76]
Ravi 2016	14.3	5.78	50	26.7	5.95	50	4.5%	-2.10 [-2.59, -1.61]
Tajalizadekhoob 2011	6	2.92	33	6.91	3.98	33	4.5%	-0.26 [-0.74, 0.23]
**Subtotal (95% CI)**			**1235**			**529**	**30.6%**	**-0.82 [-1.50, -0.14]**
Heterogeneity: Tau² = 0.77; Chi² = 111.31, df = 6 (P < 0.00001); I² = 95%								
Test for overall effect: Z = 2.35 (P = 0.02)								
**Total (95% CI)**			**1956**			**1175**	**100.0%**	**-0.32 [-0.54, -0.10]**
Heterogeneity: Tau² = 0.22; Chi² = 140.01, df = 22 (P < 0.00001); I² = 84%								
Test for overall effect: Z = 2.89 (P = 0.004)								
Test for subgroup differences: Chi² = 3.91, df = 1 (P = 0.05), I² = 74.4%								

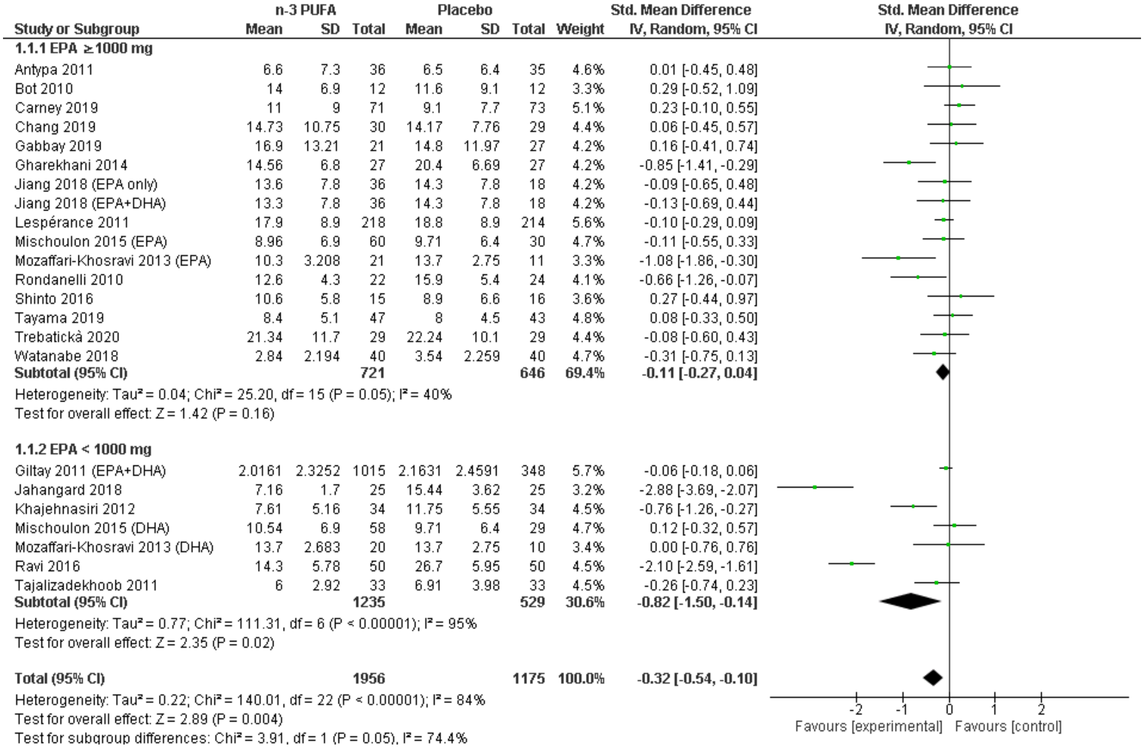

Supplementary Figure S3: Effect of n-3 PUFA on depressive symptoms by subgroup of studies with high or low EPA dosage (≥ versus <1000 mg/d)

References

Andrieu, S., S. Guyonnet, N. Coley, C. Cantet, M. Bonnefoy, S. Bordes, L. Bories, M. N. Cufi, T. Dantoine, J. F. Dartigues, F. Desclaux, A. Gabelle, Y. Gasnier, A. Pesce, K. Sudres, J. Touchon, P. Robert, O. Rouaud, P. Legrand, P. Payoux, J. P. Caubere, M. Weiner, I. Carrié, P. J. Ousset and B. Vellas (2017). "Effect of long-term omega 3 polyunsaturated fatty acid supplementation with or without multidomain intervention on cognitive function in elderly adults with memory complaints (MAPT): a randomised, placebo-controlled trial." Lancet Neurol **16**(5): 377-389.

Appleton, K. M., H. M. Sallis, R. Perry, A. R. Ness and R. Churchill (2015). "Omega-3 fatty acids for depression in adults." Cochrane Database Syst Rev(11): Cd004692.
